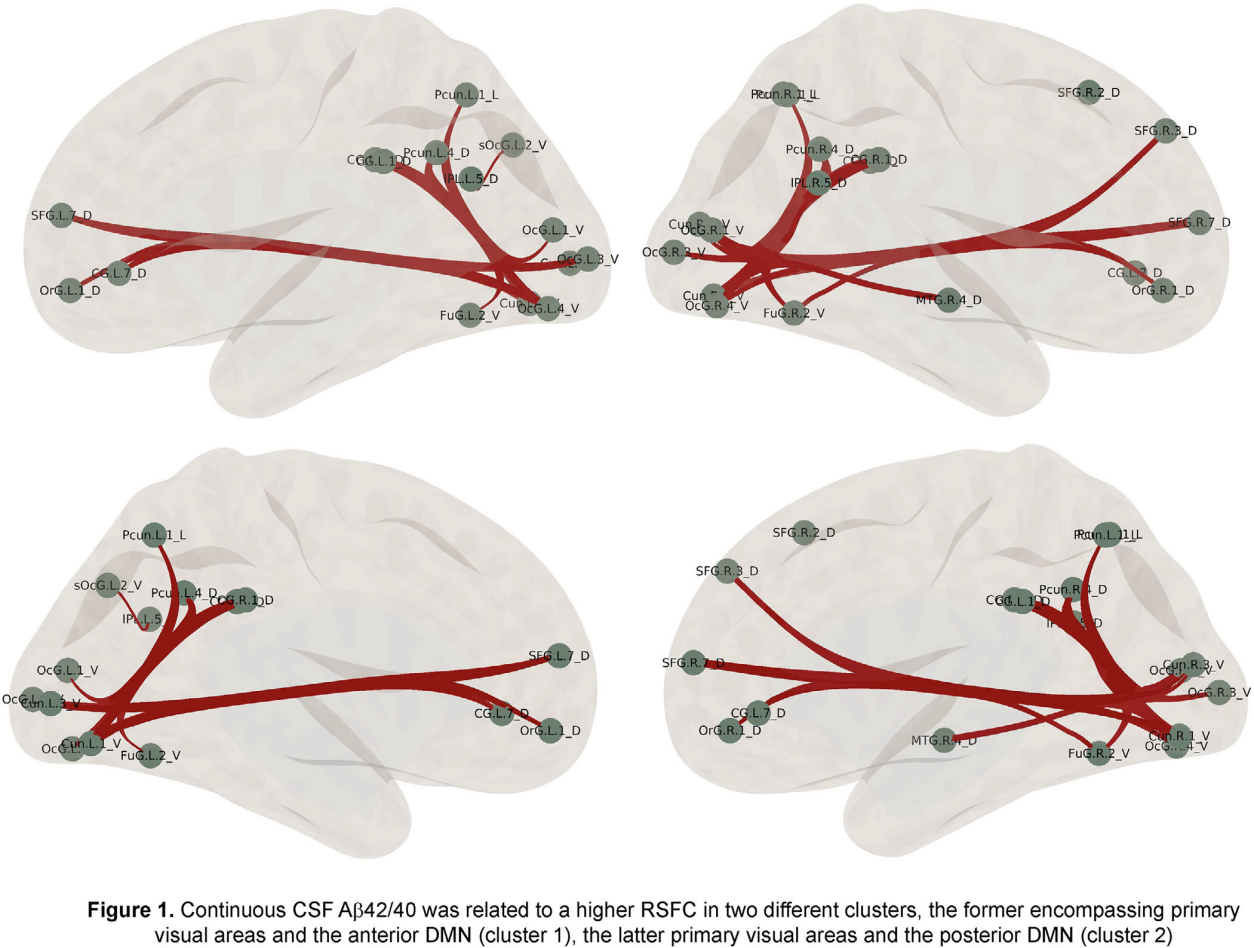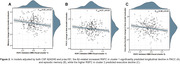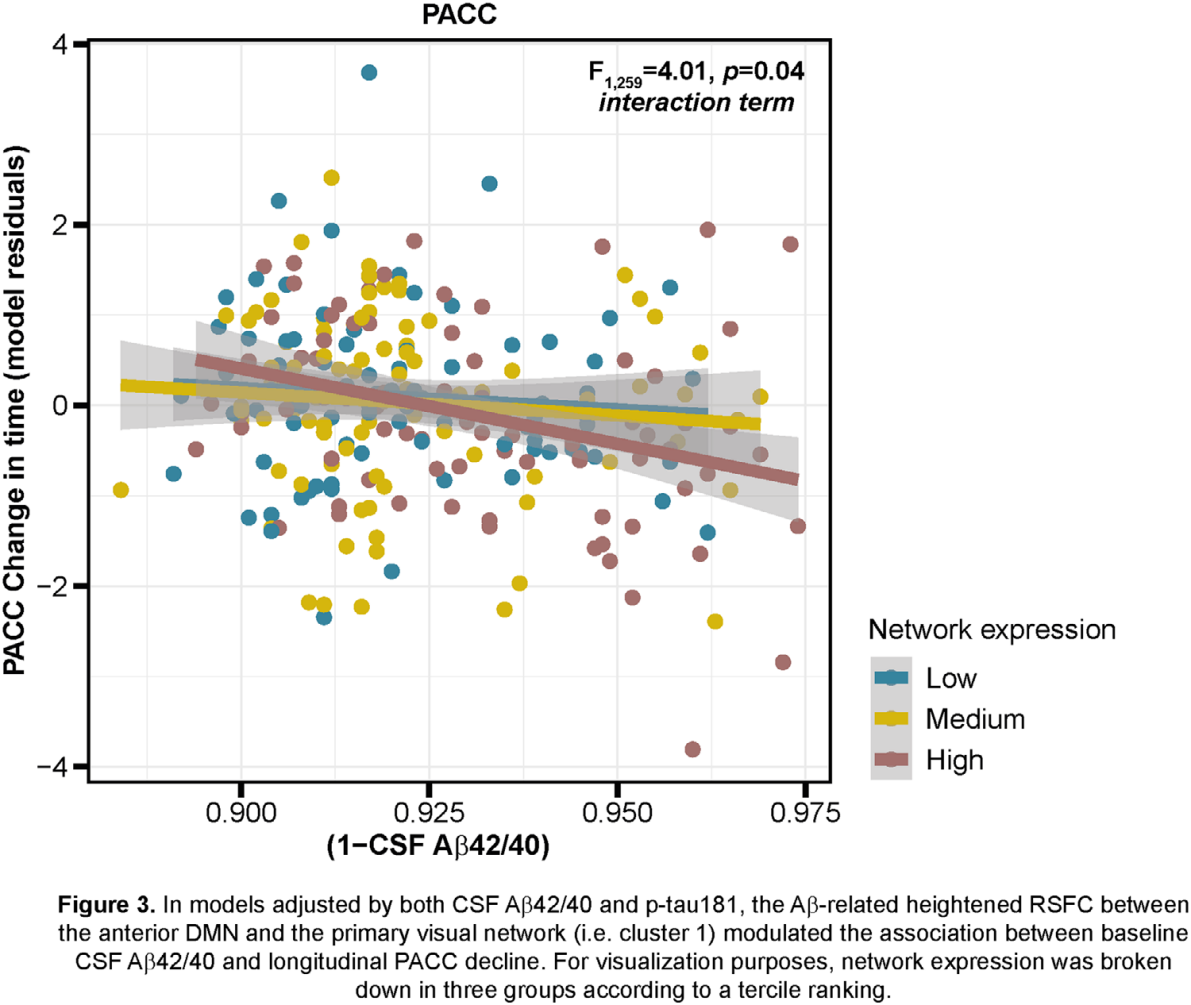# Aberrant resting‐state functional connectivity of the default‐mode network relates to cognitive decline in the earliest Alzheimer’s continuum

**DOI:** 10.1002/alz.093717

**Published:** 2025-01-09

**Authors:** Aldana Lizarraga, Michalis Kassinopoulos, José María González‐de‐Echávarri, Jordi Huguet, Gonzalo Sánchez‐Benavides, Anna Brugulat‐Serrat, Marc Suarez‐Calvet, Marta Milà‐Alomà, Kaj Blennow, Henrik Zetterberg, Gwendlyn Kollmorgen, Clara Quijano‐Rubio, Jose Luis Molinuevo, Juan Domingo Gispert, Raffaele Cacciaglia

**Affiliations:** ^1^ Barcelona?eta Brain Research Center (BBRC), Pasqual Maragall Foundation, Barcelona Spain; ^2^ Barcelona?eta Brain Research Center (BBRC), Barcelona Spain; ^3^ Centro de Investigación Biomédica en Red de Fragilidad y Envejecimiento Saludable (CIBERFES), Madrid Spain; ^4^ Hospital del Mar Research Institute (IMIM), Barcelona Spain; ^5^ Department of Veterans Affairs Medical Center, Northern California Institute for Research and Education (NCIRE), San Francisco, CA USA; ^6^ Clinical Neurochemistry Laboratory Sahlgrenska University Hospital, Mölndal Sweden; ^7^ Institute of Neuroscience and Physiology, University of Gothenburg, Mölndal Sweden; ^8^ Roche Diagnostics GmbH, Penzberg Germany; ^9^ Roche Diagnostics International Ltd., Rotkreuz Switzerland

## Abstract

**Background:**

Amyloid‐ß (Aß) pathology affects resting state functional connectivity (RSFC), even in cognitively unimpaired (CU) individuals. However, the impact of such an aberrant RSFC on cognitive decline is yet to be determined. Moreover, most prior research focused on fibrillary Aß deposition to predict RSFC, while early Aß dysmetabolism as reflected by cerebrospinal fluid (CSF) concentrations has received less attention. We assessed RSFC as a function of both CSF Aß and p‐tau in CU individuals, and further analyzed the impact of biomarker‐dependent RSFC on the longitudinal cognitive performance.

**Method:**

Analyses were conducted in 328 CU individuals from the ALFA cohort (mean age = 60.8, SD = 4.74) with available CSF Aß, p‐tau, resting‐state fMRI and longitudinal cognitive assessment (average follow‐up time = 3.35 years, SD = 0.53). CSF Aß42 and Aß40 were assessed with the exploratory NeuroToolKit, while p‐tau181 was measured with the Elecsys® Phospho‐Tau (181P) CSF immunoassay (both Roche Diagnostics International Ltd). RSFC was computed amongst 246 brain regions of the Brainnetome atlas using the CONN toolbox, selecting a cluster threshold of p<0.005. The effects of CSF biomarkers on RSFC were adjusted by age, sex, years of education and APOE‐e4 status.

**Result:**

Of the entire sample, 38.4% had positive CSF Aß42/40 markers. Low CSF Aß42/40 ratios were associated to a higher RSFC between visual areas and anterior as well as posterior subdivisions of the default‐mode network (DMN) (Fig. 1). These results survived a family‐wise error rate p‐value<0.005. High levels of CSF p‐tau were related to a higher RSFC between inferior temporal areas and the anterior DMN, as well as a reduced RSFC between visual and the somatomotor network. The Aß‐related higher RSFC significantly predicted longitudinal cognitive decline in PACC, episodic memory (EM) and executive control (EC), in models adjusted by CSF biomarkers (Fig. 2), and further modulated the association between CSF Aß42/40 and PACC longitudinal decline (Fig. 3)

**Conclusion:**

In CU individuals, CSF Aß and p‐tau affect RSFC in networks relevant for cognitive performance. Low CSF Aß42/40 was related to hyperconnectivity between the DMN and the visual system. Lack of DMN segregation as a function of CSF Aß42/40 may represent a driving mechanism of cognitive decline in the earliest Alzheimer’s continuum.